# Long COVID Syndrome Presenting as Neuropsychiatric Exacerbations in Autism Spectrum Disorder: Insights for Treatment

**DOI:** 10.3390/jpm12111815

**Published:** 2022-11-02

**Authors:** Harumi Jyonouchi, Lee Geng, Daniel A. Rossignol, Richard E. Frye

**Affiliations:** 1 Saint Peter’s University Hospital (SPUH), New Brunswick, NJ 08901, USA; 2 Rossignol Medical Center, Aliso Viejo, CA 92656, USA; 3 Rossignol Medical Center, Phoenix, AZ 85050, USA

**Keywords:** COVID-19, SARS-CoV-2, ASD, monocyte cytokine

## Abstract

COVID-19 causes not only severe respiratory symptoms, but also long-term sequelae, even if the acute-phase symptoms are minor. Neurological and neuropsychiatric symptoms are emerging as major long-term sequalae. In patients with pre-existing behavioral symptoms, such as individuals with autism spectrum disorders (ASD), the emergence of neuropsychiatric symptoms due to long COVID can be difficult to diagnose and manage. Herein, we present three ASD cases who presented with markedly worsening neuropsychiatric symptoms following COVID-19 exposure and subsequent difficulty in managing the post-COVID neuropsychiatric symptoms. Case 1 contracted SARS-CoV-2 during the early stages of the pandemic and treatment targeting COVID-19-induced immune activation was delayed. Case 2 was asymptomatic in the acute stage of a confirmed COVID-19 exposure, but still developed significant neuropsychiatric symptoms. Case 3 demonstrated a difficult course, partly due to pre-existing immune dysregulation and prior use of multiple immunomodulating agents. In cases 1 and 3 for whom serial blood samples were obtained, notable changes in the production of inflammatory and counter-regulatory cytokines by peripheral blood monocytes were observed. The presented cases illustrate the profound effects of COVID-19 on neuropsychiatric symptoms in ASD subjects and the difficulty of managing long-COVID symptoms.

## 1. Introduction

The coronavirus disease 2019 (COVID-19) pandemic caused by severe acute respiratory syndrome coronavirus 2 (SARS-CoV-2) impacted our medical system tremendously. The impact was evident in both children and in those with intellectual disability [1,2]. As the pandemic progressed, it became apparent that individuals who suffered from COVID-19 often exhibited a long-term sequela that affected multiple organ systems, referred to as ‘long COVID syndrome’ [3,4]. This condition affects both adults and children. However, the prevalence of long COVID syndrome in children is not yet well-understood, partly due to the lack of a clear-cut case definition [5,6].

Cognitive dysfunction, often referred as to ‘brain fog’, is a common manifestation of long COVID syndrome and is reported in one of every four to five post-COVID-19 patients [7]. This condition is characterized by impairments in attention, concentration, memory, speed of information processing, and executive function, and is accompanied by other neuropsychiatric symptoms (anxiety, disturbed sleep, fatigue, and depressive mood) [7,8]. Such long-term post-COVID-19 neuropsychiatric symptoms can have substantial effect on the quality of life (QOL) as well as performance at school/workplaces, creating a significant problem. 

The cognitive dysfunction that is observed in individuals suffering from long COVID syndrome resembles the syndrome of cancer-therapy-related cognitive impairment (CRCI), typically seen as a sequela of intrathecal injection of methotrexate [9]. Features of CRCI also overlap with chronic fatigue syndrome (CFS) [3,10]. Chemotherapy induced brain inflammation is mediated by microglial cells and can be created by systemic inflammation induced by low doses of lipopolysaccharide (LPS) [9] through reactive microglial cells in the white matter and monocyte/macrophage lineage cells recruited to the brain [11,12].

In children, long-term sequelae of brain dysfunction as described above may be difficult to distinguish from the psychological impact caused by the COVID-19 pandemic [5]. Indeed, COVID has increased parental stress which can result in behavioral problem [13] especially in parent of children with autism spectrum disorder (ASD) where it resulted in anxiety and depression as well as more maladaptive behaviors in the child [14]. If COVID-19-induced brain dysfunction occurs in children with intellectual disability or other pre-existing neuropsychiatric conditions, such as ASD, it becomes even harder to distinguish the COVID-19-induced new symptoms from pre-existing neuropsychiatric conditions.

ASD is a complex developmental disorder that is characterized by impaired social communications and repetitive/restrictive behaviors along with a high frequency of co-morbid conditions [15]. If children with ASD suffer from brain dysfunction as a long-term sequela of COVID-19, diagnosis and management is expected to be challenging. It is quite possible that such COVID-19-induced neuropsychiatric and neurological symptoms may be dismissed as common ASD symptoms, missing the opportunity to improve behavioral by treatment for COVID-19-induced neuroinflammation. 

This manuscript describes three ASD patients who suffered from notable and persistent changes in neuropsychiatric symptoms following COVID-19 that were proven to be challenging to manage. These cases illustrate the difficulty in treating long-term neuropsychiatric sequelae of COVID-19 infection and the need for treatment measures targeting COVID-19-induced immune activation.

## 2. Materials and Methods

### 2.1. Study Subjects

All subjects were enrolled in the institutional-review-board-approved protocol for assessment of monocyte cytokine profiles in ASD at our institution. Signed informed consent forms were obtained for all cases: parents signed the consent forms.

### 2.2. Monocyte Cytokine Profiles

Peripheral blood monocytes (PBMo) were purified as described previously [16]. PBMo were cultured overnight at a concentration of 5 × 10^5^/mL with or without stimuli of innate immunity including LPS, zymosan, CL097, and candida heat extract as a source of ß-glucan as reported previously [16]. Concentration of cytokines (IL-1ß, IL-6, IL-10, IL-23, TNF-α, sTNFRII, IL-12p40, TGF-ß, and CCL2) in the culture supernatant were measured by ELISA as reported previously [16].

## 3. Results

### 3.1. Case Presentation

**Case 1** was a twenty-five-year-old Caucasian male with ASD who has been followed in our allergy/immunology clinic. He presented with newly onset profound fatigue and generalized musculoskeletal pain accompanied by worsening fluctuation of ASD behavioral symptoms. This followed a mild respiratory infection that occurred 1 month prior to the start of the COVID-19 lockdown in the United States. He was previously diagnosed with ASD (level III), refractory epilepsy which manifested as grand mal seizures, and common variable immunodeficiency (CVID). His refractory seizures were controlled with immunomodulating agents (sirolimus and anakinra). Supplemental immunoglobulin (Ig) provided through biweekly subcutaneous (SQ) infusion helped control recurrent microbial infection and seizure clusters triggered by microbial infections. He was also on an antibiotic prophylaxis regimen (azithromycin 3x/week). This was due to a history of severe mastoiditis complicated with diffuse cholesteatoma which required multiple surgical resections. His mother also suffered from similar respiratory symptoms around the same time and started suffering from profound fatigue and ‘brain fog.’ This patient, as well as his mother, were confirmed to have had COVID-19 with the presence of SARS-CoV-2 antibodies. He had a history of having worsening behavioral symptoms (irritability, mood swing, aggression, self-injurious behaviors, and disturbed sleep) following each immune insult (typically a microbial infection), and even loss of cognitive function. His condition was thought to be associated with post-infectious neuroinflammation and was treated with various anti-inflammatory medications (non-steroidal anti-inflammatory drugs (NSAIDs), oral steroid burst, etc.) in addition to maintenance immunomodulating agents prescribed for controlling his seizures. Both he and his mother continued to suffer from persistent fatigue, ‘brain fog’, joint ache/muscle ache, and dysautonomia symptoms, which did not respond to the anti-inflammatory medications to which he responded previously. These symptoms were debilitating for him and his mother, affecting their QOL significantly. A trial of mycophenolate provided only partial symptomatic relief. With better understanding of neuropsychiatric symptoms as a long-term sequela of COVID-19 and activation pathways through SARS-CoV-2, colchicine (0.6 mg bid) was started to control such signaling pathway activation. Both he and his mother responded favorably to colchicine with substantial reduction of long COVID symptoms. Six months after starting colchicine, both his and his mother’s symptoms stabilized. However, he did not completely returned to his previous baseline, as per his mother. In his case, the similar symptoms that were manifested by his mother helped us recognize his worsening neuropsychiatric symptoms as long-term sequelae of COVID-19.

**Case 2** initially presented in our clinic at 13 years of age secondary to recurrent infections. She was diagnosed with ASD at 36 months of age due to a notable delay in development, followed by an onset of seizures at 40 months of age: her seizures were initially manifested as partial complex seizures, but became secondarily generalized. Her seizures were refractory to many anti-epileptic drugs (AEDs). She responded favorably to a modified Atkin’s diet and became seizure-free between 6 and 11 years of age. However, clusters of clinical seizures recurred after starting puberty. A high dose of valproate (375 mg at breakfast, 1,125 mg at lunch, and 2,250 mg at night) was prescribed by her neurologist at the time of initial presentation. She then started to suffer from recurrent respiratory infection around 12 years of age. Since valproate induced hypogammaglobulinemia has been reported [17], she was worked up for secondary immunodeficiency caused by AEDs. Our initial workup revealed impaired responses to the pneumococcal vaccine (PPV23). Supplemental SQ Ig controlled her recurrent respiratory infections and decreased seizure frequency. Then, she and all family members contracted COVID-19. The patient remained asymptomatic: she was fully COVID-19 vaccinated prior to the COVID-19 exposure. Her valproate dose was in the process of a slow taper starting at 15 ½ years of age. Her mother reported no notable acute symptoms including loss of taste. The patient was fully verbal and could express symptoms. Two to three weeks after contracting COVID-19, she suddenly exhibited neuropsychiatric symptoms along with marked fatigue, loss of appetite and weight loss (>20 lbs over 2 months after the onset of neuropsychiatric symptoms). Because of confirmed COVID-19 exposure prior to onset of her neuropsychiatric symptoms, she was started on celecoxib 100 mg bid for 2 weeks, followed by colchicine 0.6 mg bid. Her neuropsychiatric symptoms gradually subsided over 10-12 weeks, and colchicine was discontinued after 3 months of treatment, partly due to her gastrointestinal (GI) symptoms (mainly loose stool). She did not have any seizure clusters following COVID-19 infection. Once colchicine was discontinued, her neuropsychiatric symptoms markedly worsened and manifested as severe mood swings and temper tantrums which were controlled by increasing the dose of valproate. She continues to require celecoxib for 2 weeks intermittently for controlling flare-ups of her behavioral symptoms triggered by minor immune insults like microbial infection. Although we did not examine her monocyte cytokine profile after her SARS-CoV-2 exposure, her previous monocyte cytokine profile indicated excessive responses to innate immunity stimuli mimicking viral infection which should be effectively counter-regulated by celecoxib. Indeed, celecoxib was effective for controlling minor flare-ups.

**Case 3** is a Caucasian male patient with ASD who initially presented in our pediatric allergy/immunology clinic at 7 years of age with concerns over fluctuating behavioral symptoms, often triggered by immune insults. He was diagnosed with ASD at 3 years of age and has been unresponsive to the standard ASD treatments. Hematopoietic stem cell transplant performed at Duke University 1 year prior to his initial visit provided no symptomatic relief. Since then, he has been treated with high dose intravenous immunoglobulin (IVIg) under the diagnosis of autoimmune encephalitis (AE). However, extensive AE workup failed to detect any autoantibodies associated with AE. IVIg was later switched to SQIg with the focus of controlling post-infectious neuroinflammation. After he presented to this clinic, various treatments were tried, including multiple neurotropic medications, antioxidants, and immunomodulating agents (mycophenolate, mTOR inhibitor, etc.). Sirolimus, a mTOR inhibitor, was selected due to a new finding of vEEG abnormalities [18]. Sirolimus did provided temporary symptomatic relief, followed by a normalization of the vEEG. Extensive genetic workup including whole exome and genome sequencing (WES and WGS) did not reveal any pathogenic gene variants. Extensive immune workup focusing on neuroinflammation at another institution failed to reveal any significant results. At 9 years of age, the vEEG became abnormal again with recurrence of clinically documented epileptic activity. Anakinra was started for controlling seizure activity with temporary symptomatic relief. However, his behavioral symptoms got extremely difficult to manage. Then, all family members fell ill with COVID-19 and his behavioral symptoms worsened. A vEEG performed around that time revealed diffuse epileptic activity. He was treated as seronegative AE by other providers for several months with the use of oral steroid bolus (dexamethasone 12 mg/day × 3 every month) without symptomatic relief. Since his latest exacerbation of behavioral symptoms were likely triggered by COVID-19, baricitinib, a JAK1/2 inhibitor, was started and provided some symptomatic relief. Sirolimus which was discontinued after contracting to COVID-19, was resumed due to worsening epileptic activity after stopping sirolimus following COVID-19. His condition stabilized with baricitinib, sirolimus, and mycophenolate, although he continued to have flare-ups of neuropsychiatric symptoms following each immune insult. With a recent immune insult, a short course of colchicine helped stabilize his behavioral symptoms. Minocycline was then tried for a short course under the assumption that blocking indoleamine 2,3-dioxygenase (IDO) activity would provide additional symptomatic relief for the acute exacerbation of his neuropsychiatric symptoms apparently triggered by viral infection. It did provide symptomatic relief.

### 3.2. Changes in Monocyte Cytokine Profile before and after COVID-19

Monocyte cytokine profiles before and after contracting SARS-CoV-2 infection were available for Cases 1 and 3. In Case 1, the most notable finding was an increase in the spontaneous production of inflammatory [interleukin-1ß (IL-1ß), tumor necrosis factor-α (TNF-α), and IL-12] and counter-regulatory [IL-10 and transforming growth factor-ß (TGF-ß)] cytokines 14 mo after COVID-19 infection (time point 2). These increases returned to baseline 4 mo after starting colchicine (time point 3). In Case 3, the spontaneous production of cytokines (IL-1ß, IL-10, IL-12, and TGF-ß) prior to COVID-19 exposure was higher following a marked exacerbation of his neuropsychiatric symptoms (time point 2). Such an increase in the production of monocyte cytokines was sustained when tested again 8 months after COVID-19 exposure (time point 3). Spontaneous production of TNF-α also increased after COVID-19. Production of these cytokines in response to the stimuli of innate immunity appeared to be less affected following COVID-19 for both Cases 1 and 3 (data not shown). It has been reported that significant changes in serum levels of IL-6 in antepartum and postpartum periods is associated with a risk of ASD [19]. We have studied IL-6 production in these presented cases, but we have not seen significant changes (data not shown). This may be associated with a long half-life of IL-6 in the serum and the fact that serum IL-6 levels reflect production from multiple cellular sources, not limited to monocytes.

## 4. Discussion

It has been reported that neurological and neuropsychiatric symptoms such as ‘brain fog’ are frequent clinical manifestation of long COVID syndrome. This can occur, after recovering from the acute stage of COVID-19, even if one has suffered only mild COVID-19 symptoms [8,20]. The presented cases illustrate a variety of clinical manifestations of long-term sequelae of COVID-19 in ASD subjects for whom the acute phase of COVID-19 was either mild or asymptomatic. 

**Figure 1:**
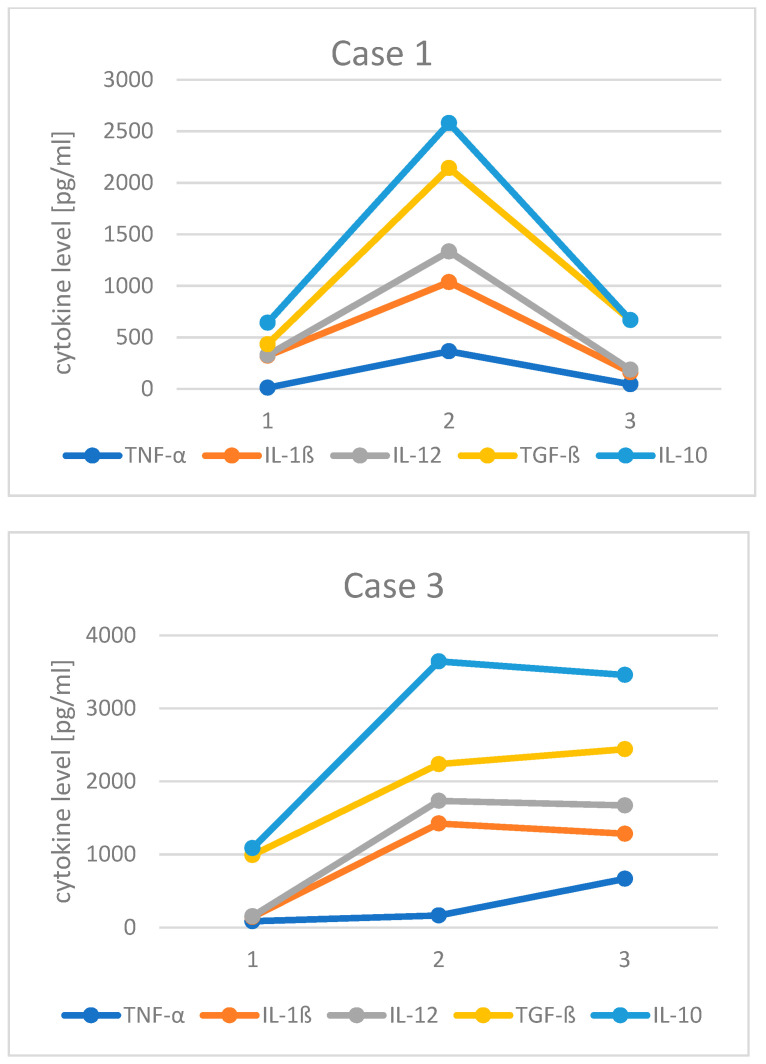
Changes in spontaneous production of TNF-α, IL-1ß, IL-12, TGF-ß, and IL-10 by purified peripheral blood monocytes in Cases 1 and 3 at different time point. **Case 1**: time point 1: before COVID-19, time point 2: after suffering from COVID-19, and time point 3: after colchicine treatment. **Case 3**: time point 1 before immune activation, time point 2: after immune activation by viral infection (not SARS-CoV-2), time point 3: after suffering from COVID-19.

A portion of ASD subjects are thought to have a component of neuroinflammation caused by complex interactions of genetic and environmental factors [21,22]. Such ASD patients are expected to have flare-ups of neuroinflammation following an immune stimulus that causes systemic immune activation; such condition is likely to occur with a viral syndrome. The emergence of COVID-19 and subsequent long-term sequelae have been shown to affect multiple organ systems, including the brain, secondary to potent and persistent immune activation [23,24,25]. Thus COVID-19 is expected to profoundly affect the state of neuroinflammation in ASD patients who have a pre-existing inflammatory component. However, this was not initially realized in the early stage of the COVID-19 pandemic. In our experience, some ASD subjects underwent extensive workups for conditions with overlapping symptoms such as AE and other autoimmune conditions. Case 1 underwent an autoimmune workup due to his persistent fatigue and musculoskeletal pain. However, in his case, since his mother had similar clinical features after contracting COVID-19 around the same time as the patient, it provides us a clue for recognizing the profound, sustained effects of COVID-19. The increase in spontaneous production of monocyte cytokines (Figure 1) also helped us recognize the persistent in vivo activation of monocyte and macrophages. At that point, we were not certain which immunomodulating medications would be helpful for controlling his condition. However, analysis of COVID-19-induced immune activation by others revealed the importance of signaling pathways of activating type I IFNs and the subsequent activation of inflammasome pathways [23,24,26]. These findings made us consider applying inflammasome blockers for controlling the dysregulated activation of monocyte/macrophage lineage cells. The persistent presence of activated T and B cells have also been reported [26], but given the fact that this patient has been treated with an mTOR inhibitor (sirolimus), a trial of blockers of activated phagocytic cells was thought to be justified. In addition, favorable response to colchicine for COVID-19 has been reported, although most of these reports focused on treating the acute phase of COVID-19 [27,28,29]. The increased spontaneous production of monocyte cytokines observed even one year after COVID-19 made us think that a trial of colchicine is justified. Indeed, he revealed favorable responses. This case may illustrate the importance of careful selection of immunomodulating agents suitable for each case. 

In Case 2, we were able to quickly recognize the possibility of long COVID-19, despite her being asymptomatic in the acute stage of COVID-19. Given her secondary hypogammaglobulinemia, likely associated with the high dose of valproate [17], and the good responses to colchicine in Case 1, she was also tried on colchicine and had a favorable clinical response. However, after discontinuation of colchicine due to GI symptoms, this patient exhibited worsening neuropsychiatric symptoms. This case also illustrates the possibility that even asymptomatic COVID-19 can still cause significant sequelae affecting the brain and lead to the possible need for a prolonged course of immunomodulating agents targeting long COVID.

Case 3 presented herein illustrated one of the most difficult cases that we encountered. In this case, the patient’s immune system was already significantly activated in vivo, as shown in Figure 1, despite a trial of multiple immunomodulating agents. COVID-19 likely further exacerbated his neuroinflammation. Given the prior use of multiple immunomodulating agents targeting various signaling pathways, after he suffered COVID-19, we attempted to block down-stream type 1 IFN signaling pathways with the use of baricitinib, a JAK1/2 inhibitor. Baricitinib has been reported to provide good immunomodulating effects in severe cases of COVID-19 and has been proposed to be a potential therapeutic agent for COVID-19 [30,31]. The patient had some clinical responses to baricitinib, but with subsequent immune activation through sick contacts (not COVID-19), its therapeutic effects were lost. Colchicine was then added to provide a broader anti-inflammatory effect for controlling post-COVID-19 immune activation. The patient seemed to benefit from a combination of colchicine and baricitinib treatment. This is an example of a case, where a combination of multiple immunomodulating agents may be required [30]. In case 1, the patient was already being treated with a mTOR inhibitor (sirolimus). This may have helped with the positive response seen from colchicine.

Due to the well-known heterogeneity of ASD, it is difficult to establish generalized practice guidelines for long COVID in ASD patients based on these three case reports. Instead, these presented cases are provided to raise awareness of the potential profound effects of COVID-19 on ASD patients and challenges for managing these patients. The presented cases may indicate a need for individualized treatment measures in patients with ASD, especially if immune mediated inflammation has a role in their behavioral dysregulation such as appears to occur in long COVID-19 syndrome.

## 5. Conclusions

In summary, the three cases presented indicate that profound, lasting effects of COVID-19 on immune activation can present as marked exacerbation of neuropsychiatric symptoms in ASD subjects, even if the acute symptoms of COVID-19 are mild or asymptomatic. Because of pre-existing difficult-to-treat ASD behaviors and limited expressive language, such late-onset, lasting effects of COVID-19 may be easily overlooked in the ASD population. A better understanding of long COVID syndrome and management options will be required in order to treat ASD patients who suffer from long COVID syndrome.

## Data Availability

The results were not submitted to any database and not available by others.

## Abbreviations Used:

AE:	autoimmune encephalitis
AEDs:	anti-epileptic drugs
ASD:	autism spectrum disorders
CFS:	chronic fatigue syndrome
COVID-19:	coronavirus disease 2019
CRCI:	cancer-therapy-related cognitive impairment
CVID:	common variable immunodeficiency
IDO:	indoleamine 2,3-dioxygenase
Ig:	immunoglobulin
IL:	interleukin
IVIg:	intravenous immunoglobulin
JAK:	Janus kinase
LPS:	lipopolysahhcaride
mTOR:	mammalian target of rapamycin
NSAIDs:	non-steroidal anti-inflammatory drugs
OCD:	obsessive compulsive disorder
PB Mo:	peripheral blood monocytes
QOL:	quality of life
SARS-CoV-2:	severe acute respiratory syndrome coronavirus 2
SQ:	subcutaneous
SPUH:	Saint Peter’s University Hospital
TGF:	transforming growth factor
TNF:	tumor necrosis factor
WES:	whole exome sequencing
WGS:	whole genome sequencing
